# Massive Infection of Lungs with Exo-Erythrocytic Meronts in European Robin *Erithacus rubecula* during Natural *Haemoproteus attenuatus* Haemoproteosis

**DOI:** 10.3390/ani11113273

**Published:** 2021-11-16

**Authors:** Carolina Hernández-Lara, Mélanie Duc, Mikas Ilgūnas, Gediminas Valkiūnas

**Affiliations:** Nature Research Centre, 08412 Vilnius, Lithuania; melanie.duc@gamtc.lt (M.D.); mikas.ilgunas@gamtc.lt (M.I.); gediminas.valkiunas@gamtc.lt (G.V.)

**Keywords:** haemosporidian parasites, *Haemoproteus*, birds, exo-erythrocytic stages, meronts, lung damage

## Abstract

**Simple Summary:**

*Haemoproteus* parasites are cosmopolitan bird pathogens belonging to the order Haemosporida (Apicomplexa). A majority of the described species are transmitted by *Culicoides* biting midges, which inject infective stages (sporozoites) in birds during blood meals. The sporozoites initiate tissue merogony, resulting in numerous merozoites, part of which penetrate red blood cells and produce blood stages (gametocytes), which are infective for vectors. The blood stages of *Haemoproteus* parasites have been relatively well-investigated, although tissue stages and patterns of their development remain unidentified in the majority of *Haemoproteus* species. Nevertheless, they often damage various organs which makes them important for bird health. This study contributes new knowledge about tissue merogony of *Haemoproteus attenuatus*, which parasitize birds of the Muscicapidae. Naturally infected European robins *Erithacus rubecula* were caught in Lithuania during autumnal migration. Parasites were identified using morphological features of gametocytes and DNA sequence analysis. Organs of infected birds were examined using histological methods. Tissue stages (meronts) were present only in the lungs, where they were numerous and markedly varied in shape, size and maturation stage. Description of meronts was provided and molecular phylogenetic analysis identified closely related lineages that could present similar exo-erythrocytic development in lungs. Lung damage caused by meronts of *H. attenuatus* and closely related lineages is worth attention due to their possible implications on a bird’s health.

**Abstract:**

*Haemoproteus* species are widespread avian blood parasites belonging to Haemoproteidae (Haemosporida). Blood stages of these pathogens have been relatively well-investigated, though exo-erythrocytic (tissue) stages remain unidentified for the majority of species. However, recent histopathological studies show that haemoproteins markedly affect bird organs during tissue merogony. This study investigated the exo-erythrocytic development of *Haemoproteus* (*Parahaemoproteus*) *attenuatus* (lineage hROBIN1), the common parasite of flycatchers (Muscicapidae). Naturally infected European robins *Erithacus rubecula* were examined. Parasite species and lineage were identified using microscopic examination of blood stages and DNA sequence analysis. Parasitaemia intensity varied between 0.8 and 26.5% in seven host individuals. Organs of infected birds were collected and processed for histological examination. Tissues stages (meronts) were seen in six birds and were present only in the lungs. The parasites were usually located in groups and were at different stages of maturation, indicating asynchronous exo-erythrocytic development. In most parasitized individuals, 100 meronts were observed in 1 cm^2^ section of lungs. The largest meronts reached 108 µm in length. Mature meronts contained numerous roundish merozoites of approximately 0.8 µm in diameter. Megalomeronts were not observed. Massive merogony and resulting damage of lungs is a characteristic feature during *H. attenuatus* infections and might occur in related parasite lineages, causing haemoproteosis.

## 1. Introduction

Avian haemosporidians (Haemosporida, Apicomplexa) are cosmopolitan parasites [1], which infect representatives of the majority of bird orders and are particularly prevalent in terrestrial bird populations [2], while, with rare exceptions, they are less often found in birds inhabiting marine and costal environments [3]. These pathogens are obligate heteroxenous. Species belonging to genera *Plasmodium*, *Haemoproteus* and *Leucocytozoon* are transmitted exclusively by blood-sucking dipterans (Insecta, Diptera). *Plasmodium* spp. are transmitted by mosquitoes (Culicidae), *Haemoproteus* (*Haemoproteus*) spp. by hippoboscid flies (Hippoboscidae), *Haemoproteus* (*Parahaemoproteus*) spp. by biting midges (Ceratopogonidae), *Leucocytozoon* (*Leucocytozoon*) spp. by simuliid flies (Simuliidae) and *Leucocytozoon* (*Akiba*) spp. by biting midges (Ceratopogonidae) [2]. Sporozoites, which are the infective stage for avian hosts, are injected during the vector’s blood meal and are transported in the blood stream to tissues of various organs where they initiate exo-erythrocytic development (meronts and/or megalomeronts). Meronts are usually relatively small (predominantly < 100 µm in length) thin-walled structures, which can be readily distinguished from megalomeronts, which are larger structures (predominantly > 100 µm in length) with a thick capsular-like wall [4]. Numerous unicellular merozoites develop in meronts and megalomeronts. Mature merozoites are released into the circulation, inhabit red blood cells and produce gametocytes, which are infective for vectors [2].

Gametocytes of haemosporidians are relatively well-studied life cycle stages, which are easy to access for microscopic examination and PCR-based research due to their presence in the peripheral blood circulation. However, tissue stages of haemosporidians are more difficult to access because this requires the dissection of bird organs and application of histopathological techniques [2]. Knowledge about exo-erythrocytic development of avian haemosporidian parasites remains scarce, particularly in *Haemoproteus* species. These haemosporidians have been formerly considered to be relatively benign avian parasites and have thus attracted insufficient attention in avian medicine and avian pathology research [4]. However, recent molecular studies combined with histopathology observations have proved that some *Haemoproteus* species cause disease and even mortality in non-accustomed avian hosts due to pathologies initiated by megalomeronts [5,6,7,8,9,10]. These findings called for further research of the exo-erythrocytic development of haemoproteids, particularly their virulence during development in specific tissues. *Haemoproteus* spp. exo-erythrocytic stages have been found in lungs, liver, spleen, kidneys, heart, brain, bone marrow, proventiculus, gizzard, caecum, tongue, intestine and skeletal muscles [4,11,12,13]. It is possible that many other organs and tissues can be involved in tissue merogony during haemoproteosis. Further studies are needed for a better understanding of the development of haemosporidian parasites in vertebrates, an issue which is directly related to bird health [4].

The aim of this study was to contribute to the characterization of the exo-erythrocytic development of *Haemoproteus attenuatus* (cytochrome *b* -cyt *b*- lineage hROBIN1) in naturally infected European robins *Erithacus rubecula*. We initiated this study due to a note in an unpublished histological observation [14], which reported the presence of meronts of *H. attenuatus* in lungs and spleen of one individual of European robin sampled during spring migration on the Baltic Sea coast. Valkiūnas [2] described this finding briefly, however, the available data about tissue merogony of *H. attenuatus* remained limited to the single observation, and the pathogen genetic lineage remained non-identified. Because *H. attenuatus* is prevalent in flycatchers of the Muscicapidae [2], we extended the observation on tissue stages in the naturally infected juvenile and adult European robins. Numerous meronts were found in lungs of parasitised birds, indicating a pattern in exo-erythrocytic development during *H. attenuatus* haemoproteosis. Phylogenetic analysis identified closely related lineages of haemoproteids inhabiting the Muscicapidae birds, suggesting a possibly similar pattern of exo-erythrocytic development of these pathogens.

## 2. Materials and Methods

### 2.1. Study Area and Sample Collection

Seven *Haemoproteus* parasite-infected European robins were caught at the Ornithological station Ventes Ragas (55°20′38.93″ N, 21°11′34.05″ E), Lithuania during autumnal migration in September 2020. Large Rybachy-like traps, zigzag and funnel traps were used for catching the birds. Among them were: 5 juveniles, 1 adult and 1 individual of unidentified age. Blood was sampled from the branchial vein and used for blood film preparation and storage in SET-buffer (0.05 M tris, 0.15 M NaCl, 0.5 M EDTA, pH 8.0) for further molecular analysis. Blood films were air dried, fixed in methanol (1 s) and stained using a 10% Giemsa solution for on-site microscopic examination following [2]. During the fieldwork, blood film microscopic examination was used to determine the presence of the parasite in the circulation, as well as preliminary species identification. SET-buffer stored blood was used later in the laboratory for parasite lineage determination (see description below). Seven *H. attenuatus*-positive birds were euthanized by decapitation, according to permits and their organs were dissected for histological examination.

### 2.2. Blood and Histological Samples

In the laboratory, blood films were stained using a 10% buffered Giemsa solution for one hour [2]. The brain, heart, intestine, kidneys, liver, lungs, pectoral muscles, spleen, and stomach were processed for histological investigation. These organs were fixed in 10% neutral formalin in the field and processed in the laboratory for long-term storage by being embedded in paraffin blocks. From each block of paraffin-embedded-organ, 4 μm sections were prepared, mounted on glass slides, air-dried and stained with haematoxylin-eosin (H&E) following standard protocols [2,15].

An Olympus BX51 light microscope equipped with an Olympus DP12 digital camera and Olympus DP-SOFT imaging software was used to examine stained blood films and histological sections. Blood films were examined for 15 min at low magnification (×400) to find infected birds. If parasites were present, 100 microscope fields were scanned at high magnification (×1000) to estimate relative infection intensity (number of parasites in 100 fields), and then parasitemia (number of parasites in 2000–10,000 erythrocytes, depending on relative infection intensity) according to [16]). Parasite species was determined at high magnification according to [2]. Histological preparations were examined at medium (×400) and high (×1000) magnification. If exo-erythrocytic meronts were found, they were examined at different magnifications (100, 200, 400 and ×1000) to identify their morphological traits and location in the organs. Exo-erythrocytic stages were then measured using ImageJ 1.53a software (National Institutes of Health, Bethesda, MD, USA, https://imagej.nih.gov/ij/USA; accessed on 10 October 2021) [17].

Voucher parasite preparations containing gametocytes (accession numbers of blood slides 49361NS-49363NS) and tissue meronts (accession numbers of histological sections of lungs 49364NS-49366NS) were deposited at Nature Research Centre, Vilnius.

### 2.3. DNA Extraction, PCR and Sequencing

DNA was extracted from blood samples stored in SET-buffer using an ammonium acetate protocol [18]. The samples were diluted to a concentration of 25 ng/μL for PCR work. A standard nested PCR protocol was applied to identify the lineage in each individual bird infected with *H. attenuatus*. The primers HaemNFI/HaemNR3 and HaemF/HaemR2, as well as the parameters of PCR, were the same as those described in the original protocol [19,20]. Positive (*Haemoproteus* sp.) and negative (nuclease-free water) controls were used as tests for possible false amplifications. PCR products were run on a 2% agarose gel to check for positive amplifications, which were sequenced from 3′ end with Big Dye Terminator V3.1 Cycle Sequencing Kit and ABI PRISMTM 3100 capillary sequencing robot (Applied Biosystems, Foster City, CA, USA). The resulted 479 bp sequences of the cytochrome *b* mitochondrial gene were checked using the SnapGene Viewer 5.2.4 software (Insightful Science, San Diego, CA, USA, www.snapgene.com; accessed on 10 October 2021) for presence of double peaks (a test for possible co-infections) and quality. The identification of lineage was carried out by BLAST-searches in MalAvi database [21] and GenBank with Megablast algorithm (www.ncbi.nlm.nih.gov/genbank/; accessed on 10 October 2021). The obtained DNA sequence information was compared with the results of the parasite microscopic identification.

### 2.4. Phylogenetic Analysis

**Figure 1:**
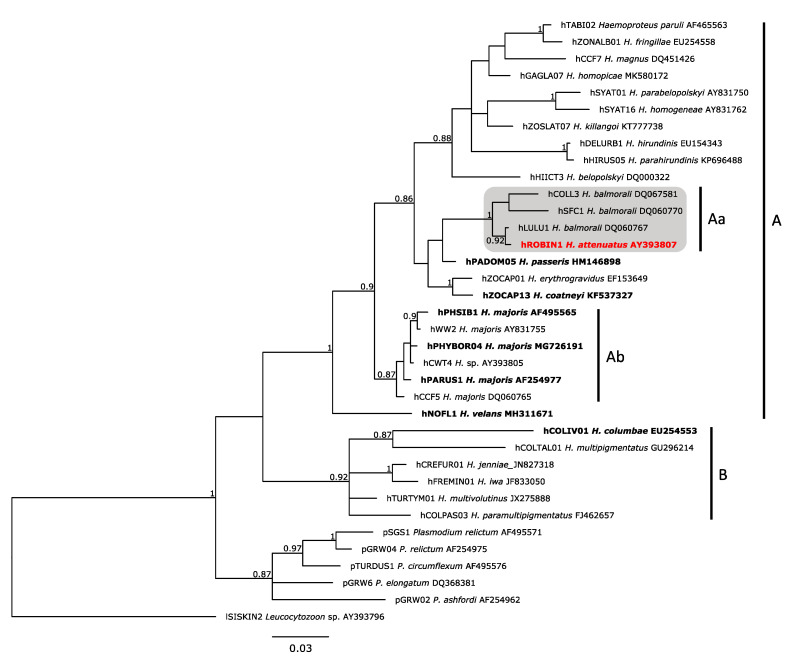
Bayesian phylogeny based on cytochrome *b* gene fragments (479 bp) of 36 lineages of avian haemosporidian parasites, including 30 lineages of *Haemoproteus*, 5 of *Plasmodium* and one of *Leucocytozoon,* which was used as outgroup. Parasite lineages were shown, followed by species names and DNA sequence GenBank accession numbers. A gray box indicates the clade containing *Haemoproteus attenuatus* (hROBIN1). Vertical bars show species of subgenera *Parahaemoproteus* (**A**) and *Haemoproteus* (**B**) as well as lineages of *Haemoproteus balmorali* (**Aa**) and *Haemoproteus majoris* (**Ab**) groups (see Discussion for explanation). *Haemoproteus* species, for which exo-erythrocytic development was formerly reported, were shown in bold font. Species investigated in this study are given in bold font and a red color. Numbers on the nodes represent posterior probabilities. Scale bar indicates the number of expected substitutions per site.

A phylogenetic tree of 36 cytochrome *b* lineages (479 bp) of avian haemosporidians was constructed using Bayesian inference in MrBayes 3.2.7 software (University of Rochester, Rochester, NY, USA; Evolutionary Biology Centre, Uppsala, Sweden) [22], which is available at the CIPRES Science Gateway [23]. Thirty lineages of *Haemoproteus,* five of *Plasmodium*, and 1 of *Leucocytozoon* (as outgroup) were used. These were DNA sequences of parasite species, which are closely related to *H. attenuatus* (the so-called parasites of a *Haemoproteus balmorali* group [2]), and other *Haemoproteus* species for which exo-erythrocytic stages have been described previously. Analysis was performed with two runs of four chains each, a 25% burn-in, 15 million generations and saving 15,000 trees. The quality of the analysis (effective sampling size, traces of the two runs, and burn-in) was examined using the Tracer 1.7.1 software (University of Edinburgh, Edinburgh, Scotland; University of Auckland, Auckland, New Zealand; University of California, Los Angeles, CA, USA) [24]. Majority rule consensus tree and the posterior probabilities were visualized using the FigTree 1.4.4 software (University of Edinburgh, Edinburgh, Scotland) [25]. Mitochondrial DNA sequences were aligned with MUSCLE [26] in Mesquite 3.61 software (The University of British Columbia, Vancouver, BC, Canada; Oregon State University, Corvallis, OR, USA) [27]. We performed the molecular analysis using a general time reversible model with gamma distribution and a proportion of invariable sites (GTR + G + I) as obtained from jModelTest 2 software (University of Vigo, Vigo, Spain; University of A Coruña, Coruña, Spain) [28]. Genetic distances between the different lineages of a *H. attenuatus* and *H. balmorali* clade (Figure 1, clade Aa) were estimated using the Jukes-Cantor model, with uniform substitution rate among sites in the Mesquite 3.61 software.

## 3. Results

### 3.1. Molecular Analysis

Single infection of *H. attenuatus* cyt *b* lineage hROBIN1 was found in the seven European robins during PCR screening. Phylogenetic analysis clustered the lineage hROBIN1 with three lineages of *H. balmorali* (hCOLL3, hSFC1 and hLULU1) in a well-supported clade (posterior probability of 1), suggesting close evolutionary relationships (Figure 1). Genetic distances between the lineages of *H. attenuatus* and *H. balmorali* clade (Figure 1, clade Aa) were small, ranging from 0.2% (hROBIN1-hLULU1) to 3.2% (hROBIN1-hSCF1).

### 3.2. Blood Stages

**Figure 2:**
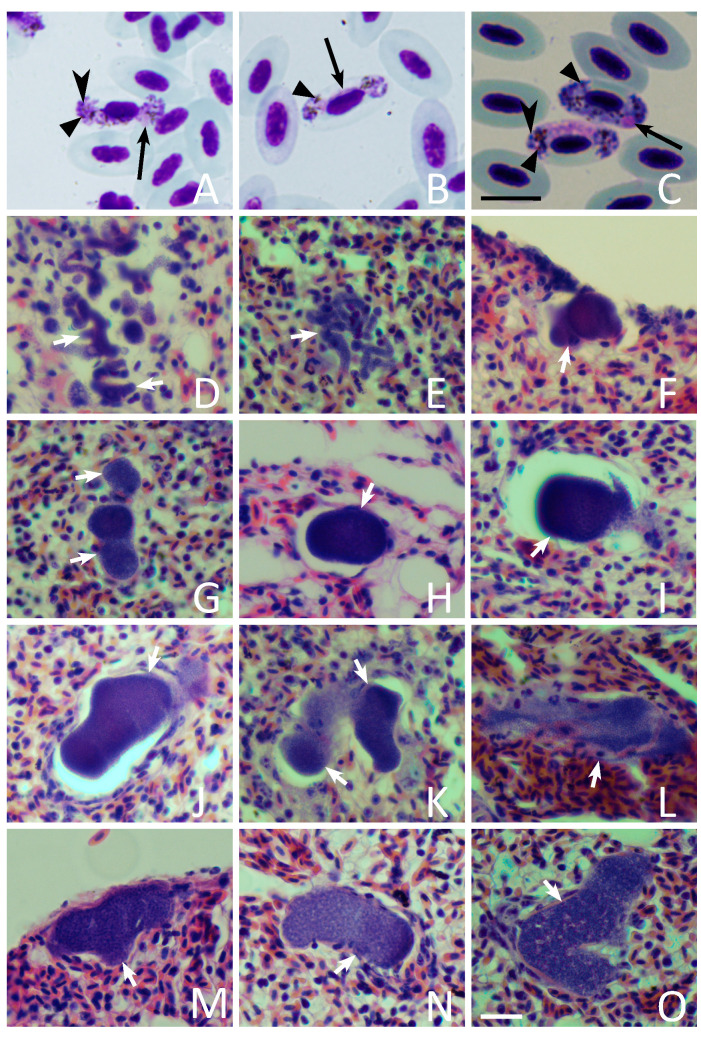
Gametocytes (**A**–**C**) and lung meronts (**D**–**O**) of *Haemoproteus attenuatus* (cytochrome *b* lineage hROBIN1) in naturally infected European robin *Erithacus rubecula*: (**A**, **B**) growing macrogametocyte (**A**) and microgametocyte (**B**); (**C**) fully grown macrogametocyte (top) and microgametocyte (bottom); (**D**,**E**) groups of numerous early developing meronts; (**F**–**O**) variously shaped maturing meronts, including those at the stage of differentiation of merozoites (**J**–**O**). Note that growing gametocytes of this parasite are markedly attenuated (**A**,**B**), which is particularly visible in microgametocyte (**B**). Prominent roundish volutin granules are well-visible in gametocytes (**A**,**C**), particularly microgametocytes, in which they gather close to the poles of the gametocytes (**C**). The largest meronts markedly push surrounding lung tissues resulting in appearance of interrupted thick envelope-like structures, which resemble a wall around some meronts (**O**), but such structures were absent around smaller meronts, which push lung cells less (**F**–**I**). Simple long black arrows—gametocyte nuclei; simple black arrowheads—volutin granules; triangle black arrowheads—pigment granules; simple short white arrows—meronts at different stages of their development. Scale bars: black—10 µm (for images **A**–**C**), white—20 µm (for images **D**–**O**).

Parasitemia intensity varied between 0.8 and 26.5% in seven examined European robins (Table 1). Observed gametocytes belonged to *H. attenuatus* (Figure 2A–C). This finding corresponded with the obtained DNA sequence information because the lineage hROBIN1 was detected. Only single infections were seen in blood films and electropherograms. Distinct characteristic features of this parasite species were readily visible (Figure 2A,C), particularly in microgametocytes. Among these features, the attenuated dumbbell-shaped growing gametocytes (Figure 2A,B) and the presence of prominent roundish volutin granules should be pointed out. Volutin granules were predominantly gathered close to the poles of gametocytes (Figure 2C).

### 3.3. Exo-Erythrocytic Stages

Megalomeronts were not seen. Meronts were found in six birds (4 juveniles, 1 adult, 1 of unidentified age; Table 1) and were present only in the lungs, where they were usually located in groups and occurred at different stages of maturation, indicating an asynchronous exo-erythrocytic development (Figure 2D–O). Meronts were not observed in one juvenile bird, in which parasitemia intensity was relatively low (0.95%). Some dissected individuals presented spleen (1 adult, 4 juveniles) and liver (2 juveniles) blackness, as well as spleen enlargement (2 juveniles; Table 1).

**Figure 3:**
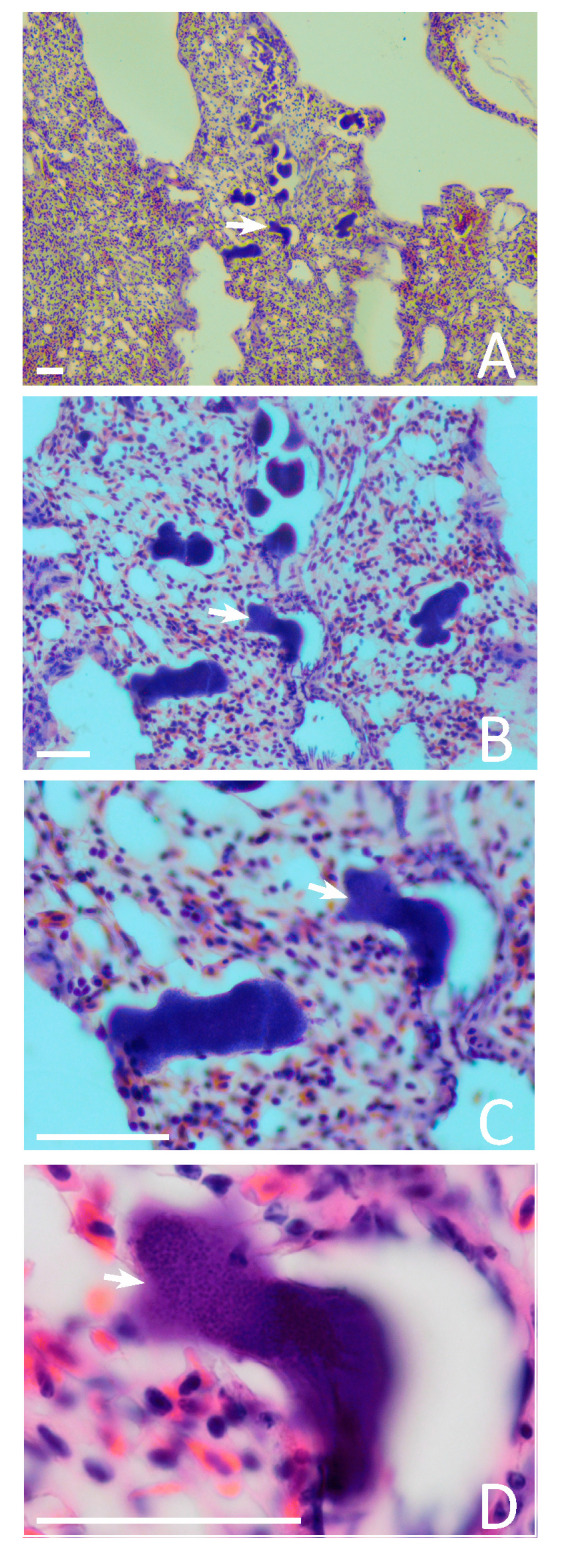
The same section of the lungs of European robin *Erithacus rubecula,* with meronts of *Haemoproteus attenuatus* (cytochrome *b* lineage hROBIN1) shown at different magnifications, ×100 (**A**), ×200 (**B**), ×400 (**C**), and ×1000 (**D**). A simple white arrow shows the same meront in this group of parasites. Location of meronts in groups is a characteristic feature of this infection (**A**,**B**). Note that meronts are markedly different in size and shapes, but the majority did not exceed 50 µm at the biggest diameter. Scale bar is 50 µm for all images.

Meronts were of markedly variable shapes and sizes. The largest one reached 108 µm at the greatest length, but the majority were smaller and usually did not exceed 50–70 µm (Figure 2D–I and Figure 3A–D). Variously shaped parasites were seen (i.e., roundish, oval, worm-like and branching; Figure 2D–O and Figure 3A–D). The elongated worm-like meronts predominated at early stages of development, suggesting that initial development occurs in the capillaries of the lungs. Available data show that the growing parasites first follow the shape of capillaries, extending along them and assuming thin elongate forms (Figure 2D,E). Then, the capillaries are blocked and deformed by growing meronts, which assume various shapes when completely grown (Figure 3A–D). The nucleus of the host cell was not seen near meronts. Vacuoles were also invisible in growing meronts, but vacuole-like spaces appeared in completely mature meronts at stage of their rupture (Figure 3D). Cytomeres were not seen at any stage of meronts growth. Mature meronts (Figure 2G–O) contained a homogenous mass of numerous roundish merozoites of approximately 0.8 µm in diameter.

Meronts were covered by a thin, often hardly visible envelope, lacking a capsular-like wall. However, largest meronts markedly pushed surrounding lung tissues, resulting in the pressure of connective fibres and the appearance of an interrupted (not entirely) thick envelope-like structure (Figure 2O). Due to interruption, this structure differed markedly from a typical entire capsular-like wall, which always develop around the megalomeronts. The interrupted thick-walled structures were absent around small meronts, which push the lung cells lightly (Figure 2F–I). In other words, the development of a thick interrupted envelope around *H. attenuatus* meronts was a function of the parasite size.

The number of meronts observed in the 1 cm^2^ section of lungs ranged from one in the least infected lungs to 100 in the most intensively infected. The largest meronts markedly pushed the surrounding lung tissues, likely resulting in a blockage of circulation in the capillaries and a deformation of alveoli (Figure 3A–D). Inflammatory reaction was not seen around the growing and maturing meronts (Figure 3A–D), but slight infiltration of blood cells was seen inside and around the largest ruptured meronts (Figure 2K,L), indicating the presence of haemorrhagic symptoms. Some cellular infiltrations were visible in the alveoli septae (Figure 2L). Furthermore, the air spaces were also seen occluded to an almost pneumonic level (Figure 2K,O), although infiltration with white blood cells was not visible.

## 4. Discussion

The main result of this study is the proof that the lungs are an important site for exo-erythrocytic development of *H. attenuatus* (hROBIN1). Meronts were observed only in the lungs. This study supported the observation by Iezhova [14] who reported morphologically similar meronts in lungs of one individual European robin infected with non-identified lineage of *H. attenuatus*. Because several *Haemoproteus* lineages have been detected in European robin (MalAvi database, Lund University, Lund, Sweden. Available online: http://130.235.244.92/Malavi; accessed on 10 October 2021), it was important to specify certain host-parasite association for this tissue stage in our study. Photographs of *H. attenuatus* meronts (Figure 2D–O and Figure 3A–D) were published for the first time. Meront morphology and localization was the same in both studies, indicating a pattern of exo-erythrocytic development of this parasite, which multiplies preferably in the lungs. Interestingly, meronts were seen in all dissected birds, except for one individual, in which parasitemia was one of the lowest (0.95%), indicating that the lung meronts may be rare and difficult to find during low parasitemia. However, the individual with the lowest parasitemia (0.8%) presented lung merogony as well as a darkened spleen.

The lineage hROBIN1 has been reported in three *Culicoides* spp. and nine bird species belonging to six families in Europe, Africa and Russia (Table 2). However, sporozoites were not observed in two *Culicoides* spp., and gametocytes of *H. attenuatus* were only seen in the blood of four bird species. In other words, presence of invasive stages (sporozoites in vectors and gametocytes in avian hosts) were not documented, meaning that some reports might be abortive infections of *H. attenuatus* [4].

Lungs have been reported as the site of meront location in several species of *Haemoproteus*, including *Haemoproteus nettionis* [42], *Haemoproteus orizivorae* [43], *Haemoproteus balearicae* [44], *Haemoproteus coatneyi* [45], *Haemoproteus columbae, Haemoproteus* sp. [46] and *Haemoproteus passeris* (see review in [2]). Interestingly, lung meronts were of similar morphology in all these parasites, and their morphology corresponded to description given in this study. Mainly, all reported lung meronts were of markedly variable sizes, shapes and developed without formation of cytomeres and capsular-like walls. DNA barcoding is available for some of these parasites. The phylogenetic analysis showed that these species are not closely related (Figure 1), probably indicating an independent evolution of the ability to inhabit lung cells in different *Haemoproteus* species.

Interestingly, Iezhova [14] reported numerous meronts of *H. attenuatus* (non-identified lineage) in lungs of an European robin sampled during spring migration, and this study found them in the same host species and organ in big numbers during autumnal migration, indicating that infected birds are present and can be detected for research during the entire period of transmission from spring to autumn in Europe. It is important to note that the presence of parasites in juvenile birds (this study) shows the local infection transmission. This information is worth attention when planning further research of this and related *Haemoproteus* infections in birds. Complete sporogony development of *H. attenuatus* (hROBIN01) occurs in the biting midge *Culicoides nubeculosus*, which might be the natural vector [29]. The same lineage was reported in *Culicoides festivipennis* and *Culicoides obsoletus*, the common biting midges in Europe (Table 2). The closely related parasite *H. balmorali* (an unidentified lineage and the lineage hSFC9) completed sporogony in *Culicoides impunctatus* [47,48]. Reports of *H. attenuatus* (hROBIN01) both in vectors and birds (Table 2) show that the transmission conditions of this infection are present in Europe.

Iezhova [14] found a single meront of *H. attenuatus* in the spleen of a naturally infected European robin, which was sampled during spring migration in May. This season corresponds to a spring relapse-period in haemosporidian parasites in Europe [2]. These data suggest that *H. attenuatus* might occasionally develop in the spleen. The latter organ might be the site of localization of persisting tissue stages, which are responsible for spring relapses, but remain insufficiently investigated in avian *Haemoproteus* parasites. Meronts in the spleen were not observed in this study, which was the autumn sample and is thus not related to spring relapse. The host–parasite association ‘*H. attenuatus* (hROBIN1) and European robin’ can be used for a deeper investigation of persistence in avian haemosporidians.

Infections detected in our study most likely corresponds to recently gained infections. Most of the infected individuals were juveniles (Table 1), meaning that they got infected on the same year of sampling. Due to the fact that only one adult bird was examined, it is not possible to make any conclusions about the influence of age of the host on merogony and pathologies found in spleen and liver, neither on the size and number of meronts or parasitemia. Nevertheless, our results suggest that even in cases of low parasitemia, alterations in spleen and liver may be present, which could have a negative implication on the host’s health.

Megalomeronts were not observed in this and Iezhova’s [14] studies, indicating that they might be absent during exo-erythrocytic development of *H. attenuatus*. The limited histological observations from natural infected birds that are available so far have reported the presence of only meronts [14,42,43,44,45,46,49,50], only megalomeronts [11,51,52,53,54,55] and both of these exo-erythrocytic stages [56,57,58,59,60,61,62,63,64,65,66,67,68,69,70,71] in different *Haemoproteus* species. A fundamental issue in biology of avian *Haemoproteus* parasites remains unresolved. Mainly, it remains unclear whether or not megalomeronts develop in all *Haemoproteus* species. In other words, it remains uncertain whether the development of both meronts and megalomeronts is an obligatory character of these parasites on a genus level. It might be that megalomeronts do not occur in some *Haemoproteus* species. It is possible that a certain sequence of occurrence during the exo-erythrocytic development (presence of meronts or megalomeronts, or both) might be a function of pathogen species or even certain host-parasite association. For example, the same isolate of *Leucocytozoon simondi*, a common haemosporidian parasite of anseriform birds, developed megalomeronts in ducks, but not in geese [2]. Megalomeronts are easy to visualise in histological sections due to their big size [11]. Meronts of some *Haemoproteus* parasites are small (close to 10 µm in diameter), contain few merozoites and are similar to meronts of *Plasmodium* spp. both by morphology and localization in organs [50], so they might be difficult to find and identify using microscopic examination of H&E stained histological sections, particularly during low intensity. Molecular diagnostic tools (chromogenic in situ hybridization) are essential in future studies of exo-erythrocytic stages, and they have already been developed [52,53]. Further targeting research is needed to better understand patterns of tissue merogony in haemosporidians. This is an important issue for current parasitology research because tissue merogony, particularly development of megalomeronts, is associated with gross pathology and is a severe, sometimes even lethal avian disease [51].

It is important to note that *H. attenuatus* (hROBIN1) is closely related to several lineages of *H. balmorali*, which also parasitize birds of the Muscicapidae (Figure 1, clade Aa). Morphological data are in accordance with these phylogenetic data. Mainly, gametocytes of these parasites share the same distinct species characters, particularly due to the presence of volutin granules of similar size, shape and location (Figure 2A–C). Recent studies show that closely related parasites, which partial cyt *b* gene sequences cluster in well-supported clades, also have tissue stages of a similar morphology and localization. For example, this is the case in different lineages of *H. majoris* (Figure 1, clade Ab), which different lineages produce megalomeronts of a similar morphology and localization in different avian hosts [11,52]. Therefore, closely related lineages of *H. attenuatus* and *H. balmorali* (Figure 1, clade Aa), which have similar gametocytes, might also present similar merogony in the lungs. In other words, when planning examination of tissue merogony of different *H. balmorali* lineages, the lungs are worth to be targeted as an important site of location of meronts first of all. This conclusion is in accordance with observation of Iezhova [14] who reported a single lung meront of non-identified lineage of *H. balmorali* in spotted flycatcher *Muscicapa striata*. Further research into a better understanding of the possible predictability of molecular phylogenies in determination of tissue merogony in haemosporidian parasites is of practical value because it might not only speed up research on this subject, but may also help to predict pathological changes in organs based solely on DNA sequence information.

This study provides limited information on the possible influence of tissue merogony on birds because it was based only on the material collected in naturally infected hosts that were euthanized (Table 1). The birds were caught in stationary traps, meaning that they were actively flying. However, the massive infection of lungs and blockage of capillaries, as well as occlusion of alveoli by tissue meronts (Figure 3), should be related to lung disfunction and a lowering of the competitive ability of intensively infected individuals. This health state is difficult to measure and correlate with bird survival without targeting experimental observations combined with field studies. Some cellular infiltrations were visible in the alveoli septae (Figure 2L). Furthermore, the air spaces were also seen occluded looking like light pneumonic degree (Figure 2K,O), although infiltration with white blood cells was not visible. High parasitemia can also hardly be neutral for the hosts, in which an enlargement and blackness of the liver and spleen was visible, indicating gross pathological changes in parenchymal organs ([2] this study). It is possible that migration behavior, which is a key feature in this host species, might be affected or even disrupted in heavily infected bird individuals. It is worth noting that former studies suggested that high *Haemoproteus* sp. parasitemia is associated with a decrease in the accumulation of migratory fat, which is the main energetic material for migrating birds [2]. Further experimental studies are needed for a better understanding of the pathologies that occur during haemoproteosis, which remains a neglected avian disease.

## 5. Conclusions

Lungs were the primary site of exo-erythrocytic development during *H. attenuatus* (hROBIN1) infection. Massive infection of lungs by meronts was determined and described in naturally parasitized birds. Megalomeronts were not observed and might not occur in this parasite during development in European robins and closely related Muscicapidae species, however, further research is needed to answer this question. Available observations and phylogenetic analysis suggested that the lineages of the *H. balmorali* group might have a similar pattern of tissue merogony, as is the case in the closely related *H. attenuatus*. Lung pathology due to the occlusion of lung capillaries and air spaces is worth attention in relation to bird health during *H. attenuatus* and related *Haemoproteus* infections.

## Figures and Tables

**Table 1:** Age, parasitemia and histological observations of seven European robins *Erithacus rubecula* infected with *Haemoproteus attenuatus* (cytochrome *b* lineage hROBIN1).

Sample No.	Age	Parasitemia (%)	Meronts in Lung Tissue	Meront Length (µm), Min–Max (*n*)	Enlarged Spleen	Darkened		Figure
						Spleen	Liver	
1	Juvenile	0.8	+	17.1–49.8 (7)	−	+	−	
2	Juvenile	0.95	−	−	+	+	−	
3	Juvenile	1.85	+	5.8–77.58 (25)	−	−	−	Figure 2D,H,I and Figure 3A–C
4	Juvenile	2.9	+	40.4–99.8 (5)	+	+	+	Figure 2M–O
5	Juvenile	23.6	+	20.6–28.5 (2)	−	+	+	
6	Adult	10.45	+	108.7 (1)	−	+	−	
7	Unknown	26.5	+	9.46–94.27 (14)	−	−	−	Figure 2E–G,J–L

**Table 2:** Hosts and locations where *Haemoproteus attenuatus* (cytochrome *b* lineage hROBIN1) have been reported.

Host Order	Host Family	Host Species	Location ^a^	Reference
Diptera	Ceratopogonidae	*Culicoides festivipennis* ^b^	Lithuania	Bernotienė et al., unpublished ^d^
		*C. obsoletus* ^b^	Lithuania	Bernotienė et al., unpublished ^d^
		*C. nubeculosus*	Lithuania	[29]
Coraciiformes	Alcedinidae	*Alcedo atthis* ^b^	Spain	Rojo et al., unpublished ^e^
Passeriformes	Certhiidae	*Certhia familiaris* ^b^	Sweden	[30]
	Acrocephalidae	*Acrocephalus schoenobaenus* ^b^	Sweden	[30]
	Sylviidae	*Sylvia communis* ^b^	Sweden	Hellgren et al., unpublished ^d^
	Muscicapidae	*Erithacus rubecula* ^c^	Bulgaria, Germany, Lithuania, Morocco, NWA, NWI, Portugal, Russia, Serbia, Spain, Sweden	[30,31,32,33,34,35,36,37]
		*Luscinia luscinia* ^c^	Lithuania, Russia, Sweden, Turkey, WGC	[30,32,34,38,39,40,41]
		*L. megarhynchos* ^c^	Bulgaria, Germany, TRC	[31,32,34]
		*Saxicola rubetra*	Nigeria, Sweden, TRC	[30,32,34]
	Turdidae	*Turdus merula* ^b^	TRC	[32]

^a^ NWA—North West Africa; NWI—North West Iberia; WGC—West Greater Caucasus; TRC—Transcaucasia. ^b^ Reports were not supported by observation of invasive stages (sporozoites in vectors or gametocytes in birds). These might be abortive infections (dead ends of transmission), particularly because gametocytes of *H. attenuatus* have never been documented in these bird species. ^c^ Co-infections with *Haemoproteus balmorali* are common in these hosts. This is an obstacle to link observed blood stages and genetic sequence information. ^d^ NCBI GenBank data. ^e^ MalAvi database data (MalAvi database. Available online: http://130.235.244.92/Malavi/; accessed on 10 October 2021).

## Data Availability

Data from this study can be available on request.
